# Left-Sided Mandibular Condylar-Ramus Deficiency Within the Craniofacial Microsomia Spectrum in a 10-Year-Old Patient: A Case Report

**DOI:** 10.7759/cureus.107975

**Published:** 2026-04-29

**Authors:** Mario Hernandez Mancillas, Vanessa P Flores Arredondo, Juan Pablo Morales, Erick M Hernández-Mancillas, Luis Fernando Jiménez Hernández

**Affiliations:** 1 General Surgery, Hospital General Instituto de Seguridad y Servicios Sociales de los Trabajadores del Estado (ISSSTE), Zacatecas, MEX; 2 General Surgery, Hospital General Zacatecas, Zacatecas, MEX; 3 Surgery, Universidad Autónoma de Durango, Durango, MEX

**Keywords:** costochondral graft, hemifacial microsomia, mandibular condyle reconstruction, omens classification, pediatric craniofacial anomaly, pruzansky-kaban classification, temporomandibular joint

## Abstract

We present the case of a 10-year-old female patient with a progressive limitation of mouth opening since early childhood, worsening at eight years of age, with difficulty tolerating solid food and impaired mastication. Physical examination revealed facial asymmetry predominantly affecting the lower third of the face, with a maximal interincisal opening of less than 8 mm and mandibular deviation toward the left during mouth opening. Computed tomography with three-dimensional reconstruction demonstrated a marked hypoplasia of the left mandibular condylar-ramus unit, a severe shortening of the ramus, and unilateral mandibular underdevelopment. Based on the clinical and imaging findings, the case was interpreted as left-sided mandibular condylar-ramus deficiency/unilateral mandibular hypoplasia within the craniofacial microsomia spectrum. Surgical management consisted of mandibular reconstruction using an autologous costochondral graft. Due to anticipated airway difficulty, orotracheal intubation was initially achieved with videolaryngoscopy, followed by temporary tracheostomy for intraoperative airway control, which was successfully decannulated seven days after surgery. A preauricular approach was used to access the temporomandibular region, and a costochondral graft harvested from the fifth rib was shaped and fixed to reconstruct the condylar-ramus unit. The patient demonstrated immediate functional improvement, with increased mouth opening observed within 24 hours postoperatively. At 12 months of follow-up, she maintained a maximal mouth opening of 16 mm, without complications and with improved tolerance to oral intake. This case documents immediate and sustained functional improvement after mandibular reconstruction in a pediatric patient with severe unilateral mandibular deficiency.

## Introduction

Craniofacial microsomia is a heterogeneous craniofacial condition characterized by the variable unilateral underdevelopment of structures that may include the mandible, temporomandibular region, ear, orbit, facial soft tissues, and facial nerve. Classification systems such as OMENS and mandibular severity grading have been used to describe this spectrum of deformity and improve phenotypic characterization [1,2]. The clinical presentation is broad, ranging from subtle mandibular asymmetry to severe unilateral mandibular deficiency with functional impairment [3,4].

Mandibular involvement is one of the most clinically relevant components of this spectrum. The Pruzansky-Kaban classification remains widely used to describe the severity of mandibular deformity, although phenotypic overlap and radiological variability may complicate strict categorization in some patients [2,3]. In more severe forms of unilateral mandibular underdevelopment, patients may present with reduced mouth opening, impaired mastication, progressive facial asymmetry, and feeding difficulty [3,4].

The management of severe unilateral mandibular deficiency in pediatric patients remains challenging. Among available reconstructive options, costochondral grafting has been used because it provides autologous tissue, structural support, and growth potential for the reconstruction of the condylar-ramus unit in growing patients [5,6]. In these patients, functional goals such as improvement in mandibular mobility and oral intake are often prioritized during early intervention [4-6]. In addition, perioperative airway planning may be particularly important in children with severe mandibular hypoplasia and restricted oral opening [7]. Historical classification systems also contributed to the conceptual framework for describing hemifacial microsomia and related mandibular deformities [8].

We present the case of a 10-year-old patient with left-sided mandibular condylar-ramus deficiency/unilateral mandibular hypoplasia within the craniofacial microsomia spectrum who underwent mandibular reconstruction with an autologous costochondral graft, with emphasis on the diagnostic considerations, surgical management, and functional outcome.

## Case presentation

Clinical history

A 10-year-old female patient with no relevant perinatal history presented with a progressive limitation of mouth opening since the age of three years. The condition worsened at eight years of age, with difficulty tolerating solid food and impaired mastication. There was no history of trauma, infection, or prior surgical intervention.

Physical examination

Facial asymmetry was evident, predominantly affecting the lower third of the face. The patient exhibited a maximal interincisal opening of less than 8 mm. Mandibular deviation toward the left side was observed during mouth opening. No facial nerve dysfunction or auricular abnormalities were identified.

Imaging findings

**Figure 1:**
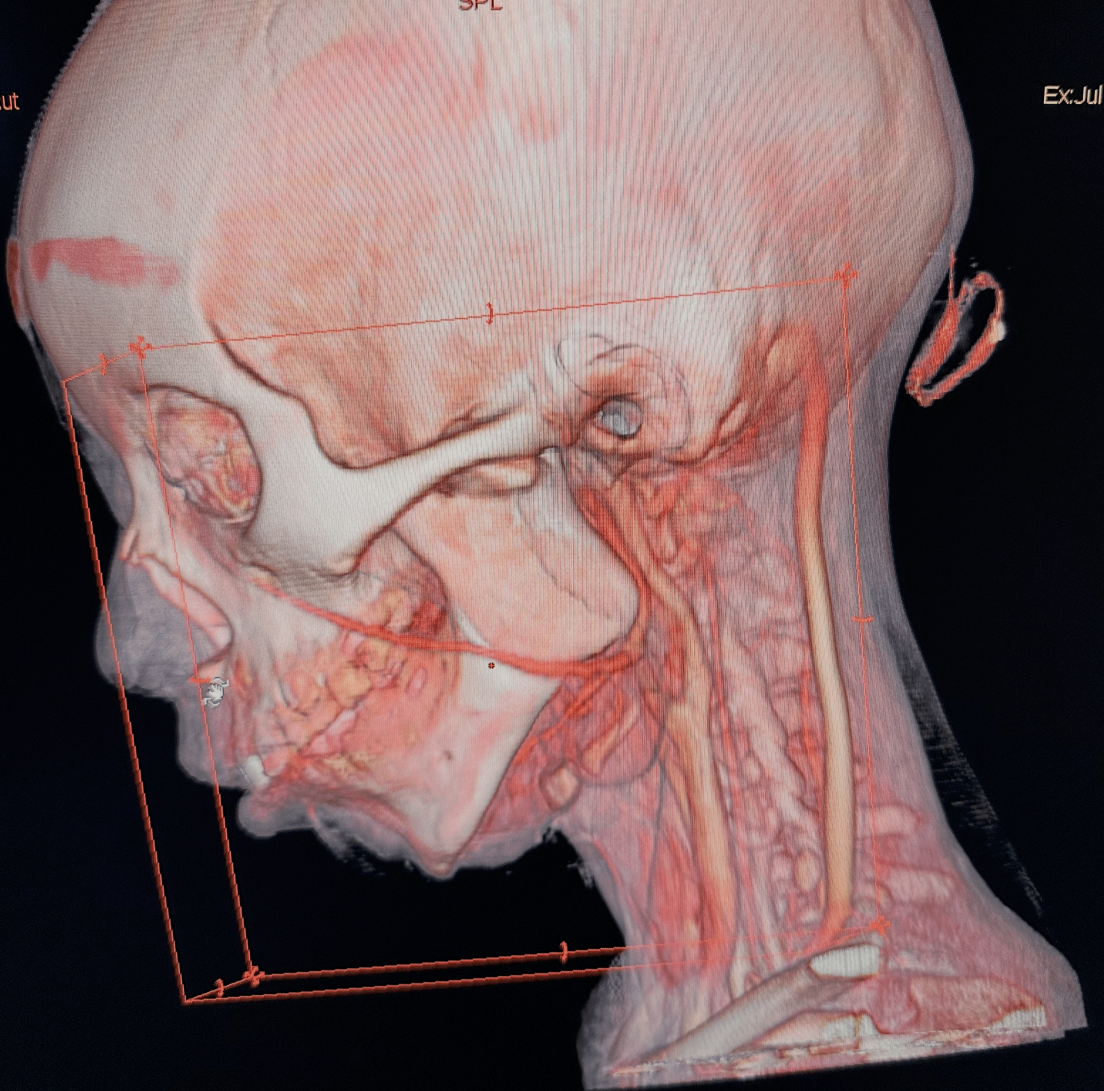
Preoperative computed tomography (CT) with three-dimensional reconstruction Preoperative three-dimensional CT reconstruction showing a marked left-sided hypoplasia of the condylar-ramus unit and a severe shortening of the mandibular ramus

Computed tomography with three-dimensional reconstruction demonstrated a marked hypoplasia of the left facial skeleton, particularly at the mandibular level, with a severe deficiency of the condylar-ramus unit and a significant shortening of the ramus height. Mandibular laterodeviation toward the affected side was also observed. These findings were interpreted as severe unilateral mandibular underdevelopment, with left-sided condylar-ramus deficiency within the craniofacial microsomia spectrum. Preoperative computed tomography with three-dimensional reconstruction is shown in Figure 1.

Diagnosis

Based on the clinical and radiological findings, the case was interpreted as left-sided mandibular condylar-ramus deficiency/unilateral mandibular hypoplasia within the craniofacial microsomia spectrum.

Surgical management

**Figure 2:**
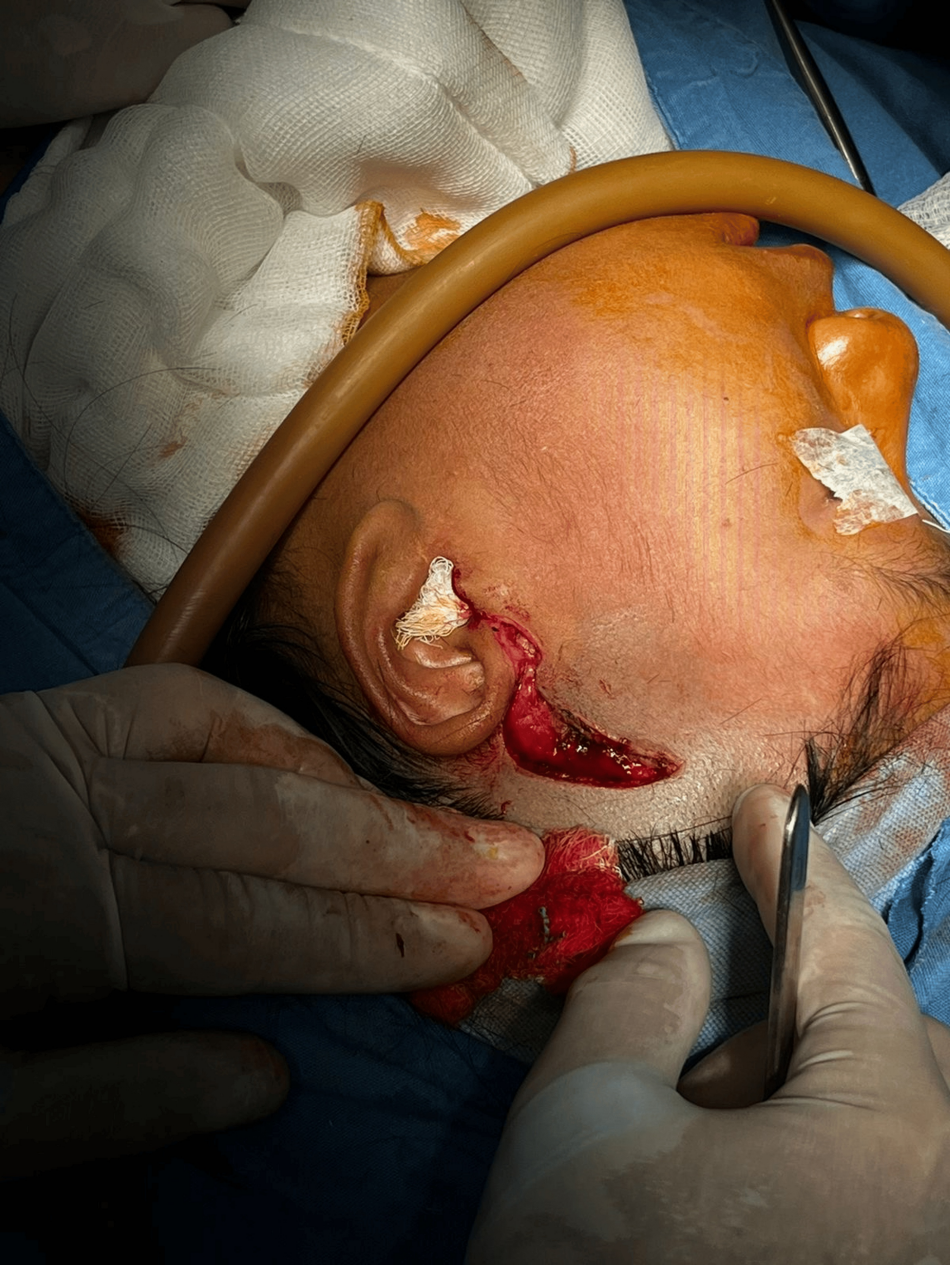
Left preauricular Al-Kayat-Bramley approach Intraoperative photograph showing the left preauricular Al-Kayat-Bramley approach used to expose the temporomandibular region

Under general anesthesia, difficult orotracheal intubation was initially achieved using videolaryngoscopy. Due to anticipated airway compromise, a temporary tracheostomy was performed for intraoperative airway control. A left preauricular Al-Kayat-Bramley approach was used to expose the temporomandibular region and the remnant of the condylar-ramus complex. Intraoperative exposure through the preauricular approach is shown in Figure 2.

**Figure 3:**
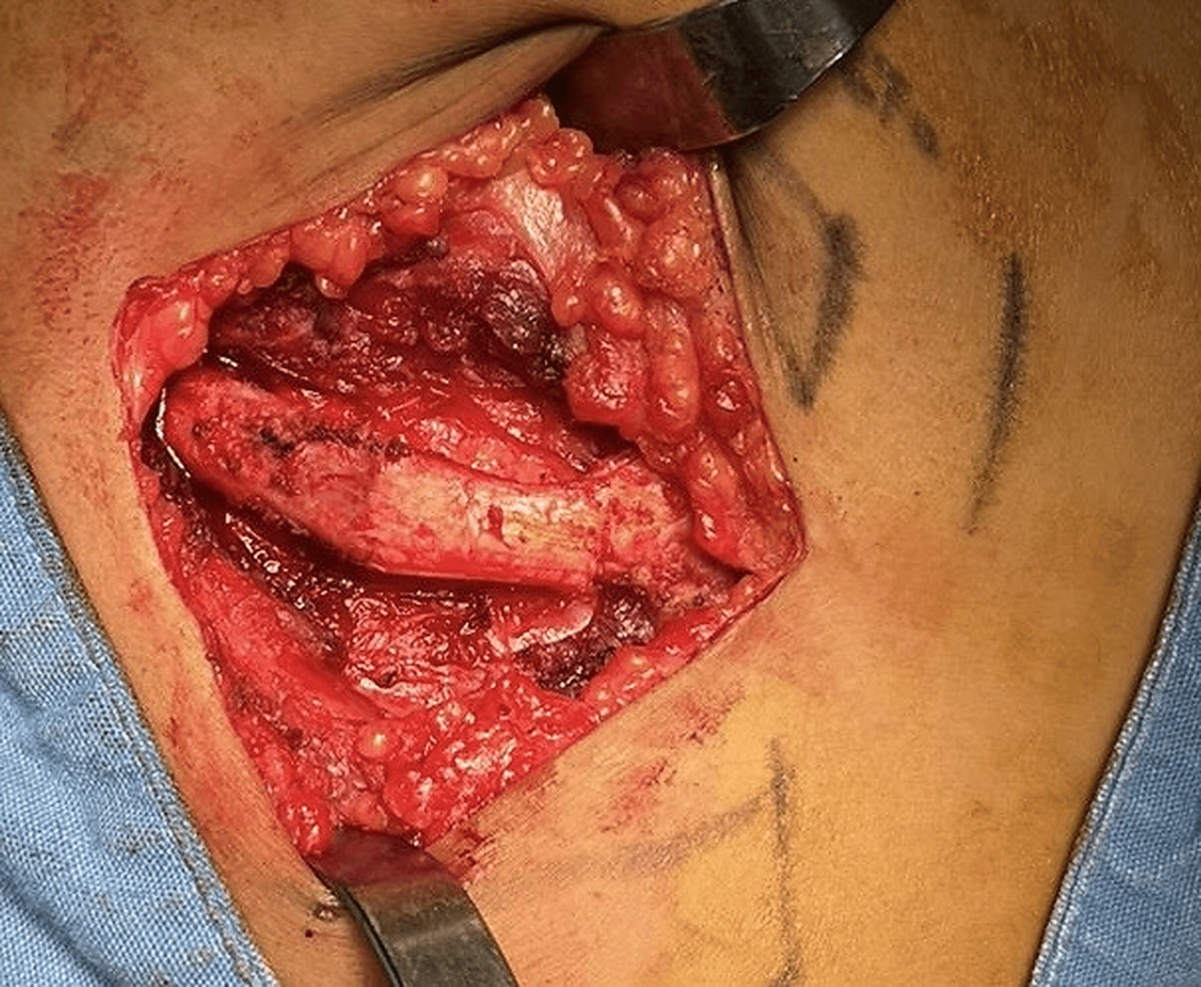
Right costal approach for costochondral graft harvest Intraoperative photograph of the right thoracic approach during the harvest of the autologous costochondral graft

**Figure 4:**
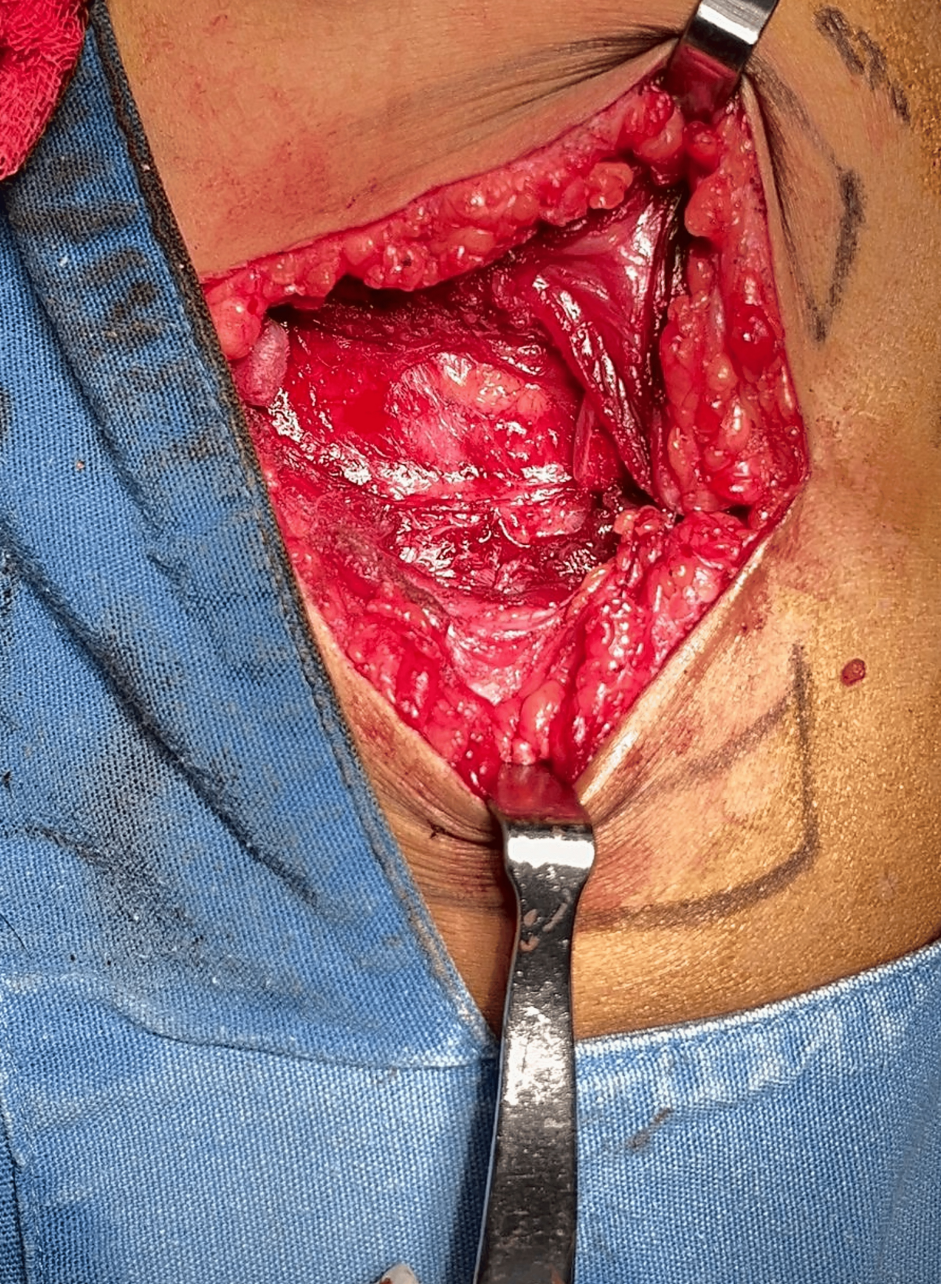
Post-harvest costal donor site Intraoperative photograph of the right costal donor site after graft harvest, showing the preservation of the underlying parietal pleura

Simultaneously, a right thoracic approach at the level of the fifth rib was performed to harvest an autologous costochondral graft, including both osseous and cartilaginous components. The rib harvest through the right costal approach is shown in Figure 3. The post-harvest costal bed is shown in Figure 4. Intraoperative video documentation demonstrated the preservation of the parietal pleura without visible pleural injury (Video 1).

**Video 1:** Intraoperative video of the right costal donor site after costochondral graft harvest Intraoperative video of the right costal donor site after costochondral graft harvest, demonstrating intact parietal pleura without visible injury

**Figure 5:**
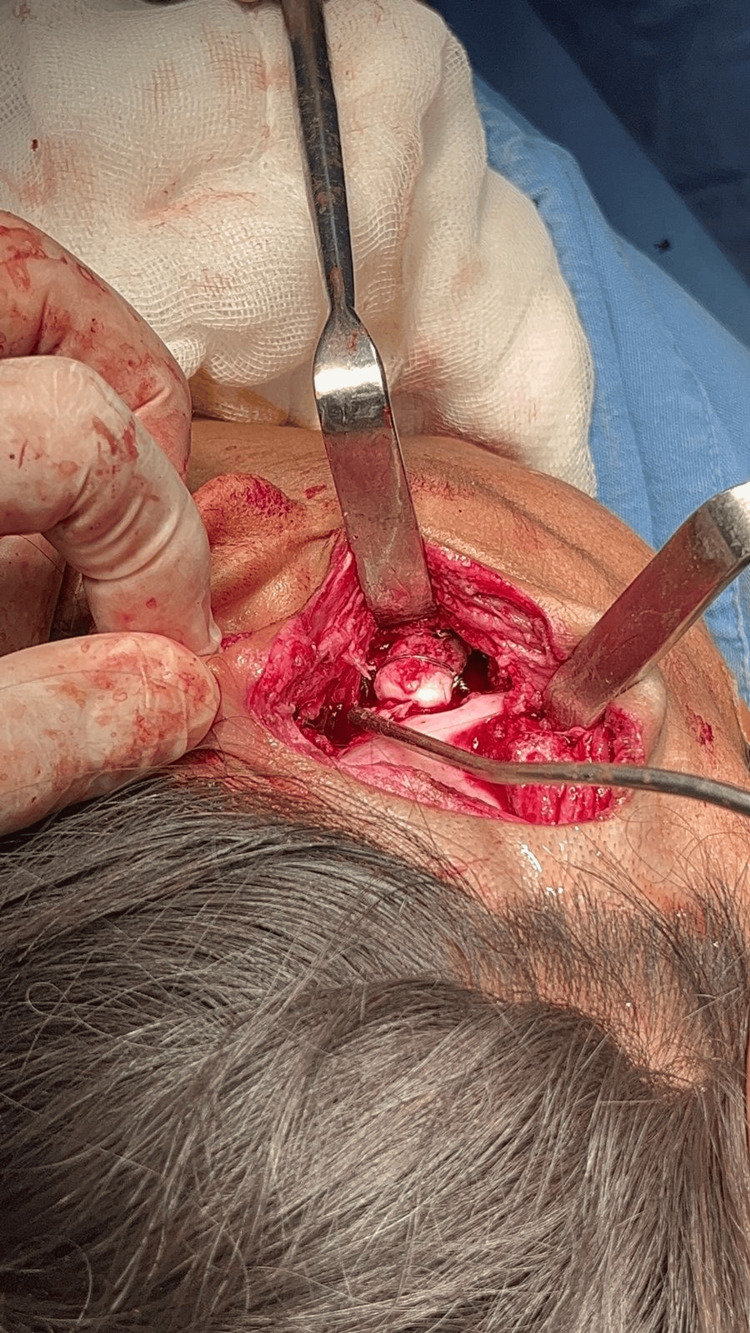
Final fixation of the autologous costochondral graft Intraoperative photograph showing the final positioning and wire fixation of the autologous costochondral graft to the residual left mandibular ramus

The graft was shaped and contoured to reconstruct the condylar-ramus unit, transferred to the recipient site, and adapted to the residual left mandibular ramus. Fixation was achieved using stainless steel wire osteosynthesis, securing the osseous portion of the graft to the mandibular remnant while positioning the cartilaginous component superiorly to recreate the condylar segment. The final intraoperative positioning and fixation of the autologous costochondral graft are shown in Figure 5. Adequate stability and alignment were confirmed intraoperatively. Closure was performed in layers at both surgical sites.

Postoperative course and outcomes

**Figure 6:**
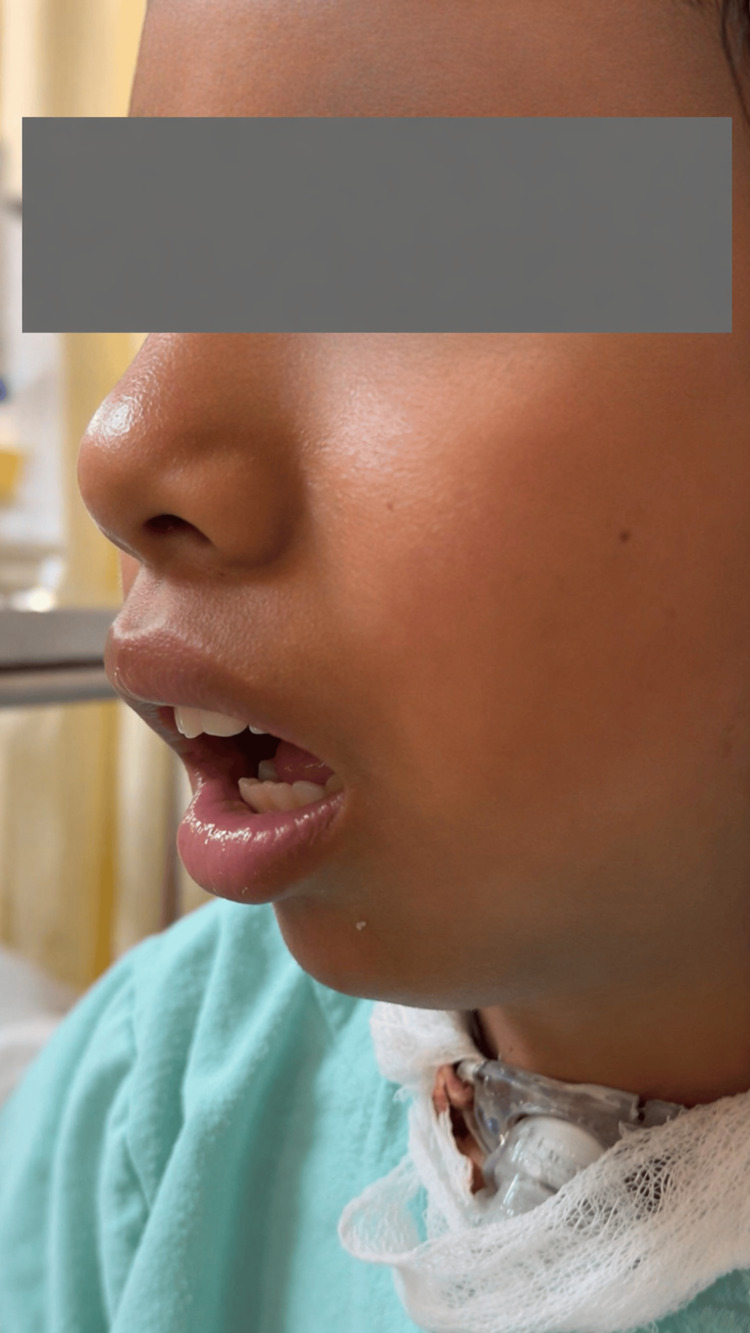
Early postoperative functional mouth opening Early postoperative clinical photograph showing improved mouth opening after reconstruction, with temporary tracheostomy still in place

The patient demonstrated immediate functional improvement, with increased mouth opening observed within the first 24 hours after surgery. The temporary tracheostomy was successfully removed seven days postoperatively without complications. At 12 months of follow-up, the patient maintained a maximal mouth opening of 16 mm, with significant improvement in oral intake and no reported complications. The treatment objective was primarily functional, with no emphasis on aesthetic correction at this stage. Early postoperative clinical assessment demonstrating functional mouth opening is shown in Figure 6.

## Discussion

Craniofacial microsomia encompasses a broad phenotypic spectrum, ranging from subtle facial asymmetry to severe unilateral mandibular deficiency with functional compromise [1-4,8]. Mandibular involvement is one of the most clinically relevant manifestations within this spectrum and may present with reduced mouth opening, masticatory dysfunction, progressive facial asymmetry, and a limitation of oral intake [2-4]. However, phenotypic overlap and radiological variability may complicate strict categorization in some patients, particularly when extracraniofacial features are limited or absent [2-4].

In the present case, the main abnormality was the severe unilateral underdevelopment of the left condylar-ramus unit, associated with functional restriction in oral opening and feeding. Although the clinical and imaging findings supported interpretation within the craniofacial microsomia spectrum, the available features did not permit unequivocal classification as classic Pruzansky-Kaban type III deformity. Likewise, the absence of auricular anomalies and facial nerve dysfunction reduced the certainty of a more definitive syndromic classification. For this reason, the case was more cautiously interpreted as left-sided mandibular condylar-ramus deficiency/unilateral mandibular hypoplasia within the craniofacial microsomia spectrum [1-4,8].

This distinction is relevant because alternative differential diagnoses should also be considered in patients with unilateral mandibular underdevelopment, including congenital condylar hypoplasia or aplasia, remote post-traumatic condylar or subcondylar growth disturbance, and, less likely, postinfectious or inflammatory sequelae involving the temporomandibular joint. In this patient, the absence of a history of trauma, infection, or prior intervention, together with the progressive limitation in mouth opening since early childhood, favored a developmental rather than acquired process. Even so, the overlap among these entities underscores the importance of cautious diagnostic wording when the phenotype is predominantly mandibular [2-4,8].

Costochondral grafting remains a widely used reconstructive option in growing patients with severe unilateral mandibular deficiency because it provides autologous tissue, structural support, and potential for continued growth [5,6]. Prior clinical series support its feasibility for mandibular reconstruction in children with severe mandibular deformity, although long-term growth behavior may be variable and sometimes unpredictable [5,6]. In the present patient, the principal treatment goal was functional restoration rather than primary aesthetic correction.

An important feature of this case was the immediate improvement in mouth opening within the first 24 hours after reconstruction, suggesting that the preoperative limitation was predominantly mechanical and directly related to the condylar-ramus deficiency. At 12 months of follow-up, the patient maintained a maximal mouth opening of 16 mm, showed improved tolerance to oral intake, and experienced no postoperative complications. Although this follow-up period is insufficient to assess definitive long-term skeletal growth, the short- and intermediate-term results support the functional utility of costochondral grafting in selected pediatric patients with severe unilateral mandibular deficiency [5,6].

Another relevant aspect of this case was perioperative airway management. Severe mandibular hypoplasia is a recognized risk factor for difficult airway control in pediatric patients with craniofacial anomalies, making preoperative planning essential. Reviews on the difficult pediatric airway emphasize the need for anticipation, appropriate equipment, and a patient-specific airway strategy [7]. In the present case, videolaryngoscopy enabled initial orotracheal intubation, and temporary tracheostomy was used for intraoperative airway control and removed seven days later without complication. This experience reinforces that airway planning should be considered a central component of management in severe mandibular deformity [7].

The donor site is also relevant when using autologous rib grafts. In this case, the graft was harvested through a right fifth-rib approach, and intraoperative visualization confirmed the preservation of the parietal pleura. Careful donor-site dissection is essential to reduce morbidity and safely obtain an adequate graft. The present report is limited by its single-case design and the absence of long-term skeletal growth analysis. Nevertheless, it highlights a practical message: in pediatric patients with severe unilateral mandibular deficiency, early reconstruction may be justified when the priority is the restoration of oral function, especially mouth opening and feeding capacity [4-6]. Historical classification systems have also contributed to the conceptual framework for understanding mandibular deformities within the craniofacial microsomia spectrum [8].

## Conclusions

Early mandibular reconstruction with an autologous costochondral graft can provide meaningful functional improvement in pediatric patients with severe unilateral mandibular hypoplasia. In this case, the reconstruction of the condylar-ramus unit resulted in immediate postoperative improvement in mouth opening, sustained functional benefit at 12 months, and no complications. This case emphasizes that, in severe unilateral mandibular deficiency, treatment should prioritize the restoration of oral function and careful perioperative airway planning.
